# Dynamical instability of the electric transport in superconductors

**DOI:** 10.1038/s41598-018-32302-8

**Published:** 2018-09-20

**Authors:** Lei Qiao, Dingping Li, Svetlana V. Postolova, Alexey Yu. Mironov, Valerii Vinokur, Baruch Rosenstein

**Affiliations:** 1 School of Physics, Peking University, Beijing, 100871 China; 2 Collaborative Innovation Center of Quantum Matter, Beijing, China; 3 Institute for Physics of Microstructures RAS, GSP-105, Nizhny Novgorod, 603950 Russia; 4 A. V. Rzhanov Institute of Semiconductor Physics SB RAS, Novosibirsk, 630090 Russia; 5 Department of Physics, Novosibirsk State University, Novosibirsk, 630090 Russia; 6 Argonne National Laboratory, Materials Science Division, Lemont, IL 60439 USA; 7 Electrophysics Department, National Chiao Tung University, Hsinchu, 30050 Taiwan Republic of China; 8 Physics Department, Bar-Ilan University, 52900 Ramat-Gan, Israel

## Abstract

We develop a nonlinear theory of the electronic transport in superconductors in the framework of the time-dependent Ginzburg-Landau (TDGL) equation. We utilize self-consistent Gaussian approximation and reveal the conditions under which the current-voltage *V*(*I*) dependence (*I*–*V* characteristics) acquires an S-shape form leading to switching instabilities. We demonstrate that in two-dimensions the emergence of such an instability is a hallmark of the Berezinskii-Kosterlitz-Thouless (BKT) transition that we have detected by transport measurements of titanium nitride (TiN) films. Our theoretical findings compare favorably with our experimental results.

## Introduction

A panoply of nonlinear and instability effects in electronic transport in superconductors includes instabilities stemming from overheating and vortex contraction effects and switching instabilities triggered by vortex depinning-like instabilities^1^. The signatures of these instabilities are the related S-shaped *I*(*V*) dependences. The common view assign the emergence of S-like *I*(*V*) curves to the classical overheating of an electron gas by the external field^1–4^. If a bottleneck of the relaxation process is the energy transfer from electronic system to the thermal bath, the effective temperature of electrons *T*_*e*_ becomes higher than the temperature of phonons *T*_*ph*_. A steep temperature dependence of the resistance *R*(*T*) is viewed as a signal that the system in question has a tendency towards an instability^2^. In particular, thermal instabilities are likely to develop near the superconducting transition temperature *T*_*c*_. The reason is the sharp *R*(*T*) dependence in the critical vicinity of *T*_*c*_ primarily due to divergent Aslamazov-Larkin contribution $\sim 1/\,{\rm{l}}{\rm{n}}(T/{T}_{c})$. The applicability of the above model to superconducting films was examined in work^5^ and it was revealed, in particular, that the power-balance equation^3,4^ correctly describes the behavior of the *I*-*V* curves of thin superconducting films at *T* > *T*_*c*_. At these temperatures, however, bistability is absent in films and develops only at *T* < *T*_*c*_.

At the same time, the early classical works by Gorkov^6^ and Masker, Marcelja and Parks^7^ suggested the existence of the intrinsic *I*-*V*, purely superconducting instabilities caused by the superconducting (SC) fluctuations rather than the overheating effects. In one dimension the analysis presented by Tucker and Halperin^8^ utilized the dynamical Hartree - Fock approximation and indicated the possibility for instability. Similar instability was found in^9^ for 1D TDGL. Experiments on quasi-1D and 2D superconducting systems revealed instability effects^10–13^, where voltage jumps were found in the current driven settings^14–17^. Moreover, experiments on systems with Cooper pair chanel revealed that while the high-resistive branches of the *I*(*V*) curves may fairly well fit the overheating behavior, the latter fails to describe the low-resistance branches^18^. This calls for a thorough understanding of the role of the intrinsic SC fluctuations-induced instabilities not related to overheating effects and the associated with them emergence of hot spots and/or moving heating domains. Our paper takes up on the task and expands the self-consistent approach following refs^19–23^ that provides a quantitative description of transport instabilities in superconductors.

## Thermal Fluctuations and Electric Field in TDGL Equation

### The model

The GL free energy has a standard form^23,24^:

1 $${F}_{GL}=A\,\int {d}^{D}{\bf{r}}[\frac{{\hslash }^{2}}{2{m}^{\ast }}|\nabla {\rm{\Psi }}{|}^{2}+\alpha (T-{T}_{c0})|{\rm{\Psi }}{|}^{2}+\frac{b}{2}|{\rm{\Psi }}{|}^{4}],$$

with Ψ being the order parameter, *D* being the dimension of the system and *A* being either the cross section for the wire (for the 1D case) or the thickness of the film. In the GL potential term, *T*_*c*0_ is the mean-field critical temperature. The GL coefficients define as usual the superconducting coherence length *ξ*^2^ = ℏ^2^/(2m^*^αT_*c*0_) and the London penetration depth *λ*^2^ = *bc*^2^m^*^/(16*πe*^2^*αT*_*c*0_). The relaxation dynamics of the superconducting order parameter in the presence of electric field *E* is described by the gauge-invariant TDGL equation^22^ with the Langevin white noise:

2 $${{\rm{\Gamma }}}_{0}^{-1}(\frac{\partial }{\partial \tau }-i\frac{{e}^{\ast }\phi }{\hslash }){\rm{\Psi }}=-\,\frac{1}{A}\frac{\delta {F}_{GL}}{\delta {{\rm{\Psi }}}^{\ast }}+\zeta ({\bf{r}},\tau ).$$

Here the order parameter relaxation time is given by ${{\rm{\Gamma }}}_{0}^{-1}={\hslash }^{2}\gamma /(2{m}^{\ast })$, where the inverse diffusion constant *γ*/2, controls the time scale of the dissipation dynamical processes. The scalar potential for constant homogeneous electric field applied along the *x* axis is *φ* = −*Ex*. The thermal forces, which induce the thermodynamical fluctuations, satisfy the fluctuation-dissipation theorem

3 $$\langle {\zeta }^{\ast }({\bf{r}},\tau )\zeta ({\bf{r}}^{\prime} ,\tau ^{\prime} )\rangle =\frac{2T}{A{{\rm{\Gamma }}}_{0}}\delta ({\bf{r}}-{\bf{r}}^{\prime} )\delta (\tau -\tau ^{\prime} \mathrm{).}$$

The electric current density includes two components, **J** = **J**_*n*_ + **J**_*s*_, where **J**_*n*_ = *σ*_*n*_**E** is the current density contributed by the Ohmic normal part and **J**_*s*_ is fluctuation supercurrent density given by

4 $${{\bf{J}}}_{s}=\frac{i{e}^{\ast }\hslash }{2{m}^{\ast }}({{\rm{\Psi }}}^{\ast }\nabla {\rm{\Psi }}-{\rm{\Psi }}\nabla {{\rm{\Psi }}}^{\ast }).$$

### Characteristic scales and dimensionless variables

We will be measuring the distances in the units of the coherence length *ξ*, the time in the units of the GL “ relaxation” time^22,23^ *τ*_*GL*_ = *γξ*^2^/2. The fluctuation strength is characterized by the parameter

5 $$\omega =2\pi ({T}_{{\rm{c}}0}{e}^{\ast 2}{\lambda }^{2}/A{c}^{2}{\hslash }^{2}{\xi }^{D-2}),$$

where *D* is dimensionality of the system; *A* is the thickness for *D* = 2 or the cross-section area for *D* = 1. The order parameter is normalized by Ψ^2^ = (2*αT*_*c*0_/*b*)*ψ*^2^ and electric field by $E={E}_{GL} {\mathcal E}$, where

6 $${E}_{GL}=2\hslash /\gamma {e}^{\ast }{\xi }^{3}.$$

The TDGL Eq. (2), written in dimensionless units reads,

7 $$({D}_{\tau }-\frac{1}{2}{\nabla }^{2})\psi +\frac{t-1}{2}\psi +{|\psi |}^{2}\psi =\bar{\zeta },$$

where *t* ≡ *T*/*T*_*c*0_, ${D}_{\tau }=\frac{\partial }{\partial \tau }+i {\mathcal E} y$ and *ζ* = $\bar{\zeta }$(2*αT*_*c*0_)^3/2^/*b*^1/2^, the white noise correlation takes a dimensionless form:

8 $$\langle {\bar{\zeta }}^{\ast }({\bf{r}},\tau )\bar{\zeta }({\bf{r}}^{\prime} ,\tau ^{\prime} )\rangle =2\omega t\delta ({\bf{r}}-{\bf{r}}^{\prime} )\delta (\tau -\tau ^{\prime} \mathrm{).}$$

Finally, the dimensionless current density **j**_*s*_ = **J**_*s*_/*J*_*GL*_, with *J*_*GL*_ = *cH*_*c*2_*ξ*/2*πλ*^2^ as the unit of the current density, is

9 $${{\bf{j}}}_{s}=\frac{i}{2}({\psi }^{\ast }\nabla \psi -\psi \nabla {\psi }^{\ast }).$$

Finally, the GL conductivity is

10 $${\sigma }_{GL}\equiv {J}_{GL}/{E}_{GL}={c}^{2}\gamma {\xi }^{2}/4\pi {\lambda }^{2}.$$

## The Self - Consistent Approximation Calculation of the I-V Curve

In what follows we will use the Hartree - Fock type self-consistent Gaussian approximation (SCGA)^25–28^ used in the past to calculate fluctuation contribution to magnetization^29^, Nernst effect^25^ and conductivity above *T*_*c*_^30^.

### Dynamical Gaussian approximation

The TDGL in the presence of the Langevin white noise, Eq. (7), is nonlinear, so cannot generally be solved. Since we will need only the thermal averages of quadratic in *ψ* quantities, like the superfluid density and the electric current, a sufficiently simple and accurate approximation (similar in nature to the Hartree-Fock approximation in the fermionic models) is the gaussian approximation^25,28,29^. The nonlinear |*ψ*|^2^*ψ* term in the TDGL Eq. (7) is approximated by a linear one 2〈|*ψ*|^2^〉*ψ* (there are two possible contractions between ψ^*^, *ψ* in |*ψ*|^2^*ψ*, see discussion of this point in^19–21^):

11 $$({D}_{\tau }-\frac{1}{2}{\nabla }^{2}+\frac{t-1}{2}+2\langle {|\psi |}^{2}\rangle )\psi ({\bf{r}},\tau )=\bar{\zeta }({\bf{r}},\tau \mathrm{).}$$

For stationary homogeneous processes considered here, the superfluid density 〈|*ψ*|^2^〉 is just a constant. Now it takes a form,

12 $$[{D}_{\tau }-\frac{1}{2}{\nabla }^{2}+\varepsilon ]\psi ({\bf{r}},\tau )=\bar{\zeta }({\bf{r}},\tau ),$$

where the excitations energy gap^23^ is,

13 $$\varepsilon =-\,\frac{1-t}{2}+2\langle {|\psi |}^{2}\rangle .$$

The solution therefore can be written via the Green’s function,

14 $$\psi ({{\bf{r}}}_{1},{\tau }_{1})=\int d{{\bf{r}}}_{2}\int d{\tau }_{2}G({{\bf{r}}}_{1},{\tau }_{1};{{\bf{r}}}_{2},{\tau }_{2})\bar{\zeta }({{\bf{r}}}_{2},{\tau }_{2}){\rm{.}}$$

Then the superfluid density, using the noise correlator, Eq. (8), can be expressed via the Green’s function as,

15 $$\langle {|\psi ({{\bf{r}}}_{1},{\tau }_{1})|}^{2}\rangle =2\omega t\int d{{\bf{r}}}_{2}\int d{\tau }_{2}{G}^{\ast }({{\bf{r}}}_{1},{\tau }_{1};{{\bf{r}}}_{2},{\tau }_{2})G({{\bf{r}}}_{1},{\tau }_{1};{{\bf{r}}}_{2},{\tau }_{2}),$$

and is a function of the parameter *ε* which is determined self consistently by Eq. (13).

### Green’s function for a homogeneous constant electric field

To calculate the response of the system, one needs the Green’s function in the presence of electric field:

16 $$G({{\bf{r}}}_{1},{{\bf{r}}}_{2},\tau )=\theta (\tau )\frac{1}{{(2\pi \tau )}^{D\mathrm{/2}}}\exp [-\varepsilon \tau -{ {\mathcal E} }^{2}\frac{{\tau }^{3}}{24}-\frac{i {\mathcal E} }{2}\tau ({x}_{1}+{x}_{2})-\frac{{({{\bf{r}}}_{1}-{{\bf{r}}}_{2})}^{2}}{2\tau }]{\rm{.}}$$

The invariance with respect to the time translations is already taken into account by setting *τ* = *τ*_1_ − *τ*_2_. Using these expressions, the superfluid density of Eq. (15) takes a form,

17 $$\langle {|\psi ({\bf{r}},\tau )|}^{2}\rangle =\frac{\omega t}{{2}^{D-1}{\pi }^{D\mathrm{/2}}}{\int }_{0}^{\infty }\frac{d\tau }{{\tau }^{D\mathrm{/2}}}\exp [-2\varepsilon \tau -{ {\mathcal E} }^{2}\frac{{\tau }^{3}}{12}].$$

The integrand in Eq. (17) is divergent as 1/*τ* when *τ* → 0 when *D* > 1. The cutoff *τ*_*cut*_ is thus required to account for the inherent UV divergence of the Ginzburg-Landau theory and it will be addressed below.

Finally the gap equation assumes the form

18 $$\varepsilon =-\,\frac{1-t}{2}+\frac{\omega t}{{2}^{D-2}{\pi }^{D\mathrm{/2}}}{\int }_{{\tau }_{cut}}^{\infty }\frac{d\tau }{{\tau }^{D\mathrm{/2}}}\exp [-2\varepsilon \tau -{ {\mathcal E} }^{2}\frac{{\tau }^{3}}{12}].$$

After (numerical) solution for the energy gap *ε*, we turn to calculation of the supercurrent. While the upper limit of the integration in Eq. (18) is safe (both terms in exponent are positive), the lower limit (UV) depends on dimensionality.

In the paper [^25^], it was shown that *τ*_*cut*_ in time dependent Ginzburg Landau and the energy cutoff Λ in static Ginzburg Landau theory are related by

19 $${\tau }_{cut}=\frac{{\hslash }^{2}}{2{m}^{\ast }{\xi }^{2}{\rm{\Lambda }}{e}^{{\gamma }_{E}}}$$

where *γ*_*E*_ is Euler constant and Λ is the energy cutoff^25,29^. Our calculation show that taking value *τ*_*cut*_ from 0.1 to 10, the physical quantities is essentially unchanged and is taken as *τ*_*cut*_ = 1 in what follows.

### The electric current density

The dimensionless supercurrent density along the electric field direction *x*, defined by Eq. (9), expressed via the Green’s functions is

20 $$\langle {j}_{x}^{s}\rangle =i\omega t\,\int d{{\bf{r}}}_{2}d\tau ^{\prime} {G}^{\ast }({{\bf{r}}}_{1},{{\bf{r}}}_{2},\tau -\tau ^{\prime} )\frac{\partial }{\partial x}G({{\bf{r}}}_{1},{{\bf{r}}}_{2},\tau -\tau ^{\prime} )+c{\rm{.}}c$$

Performing the integrals, one obtains,

21 $$\langle {j}_{x}^{s}\rangle =\frac{\omega t {\mathcal E} }{{2}^{D}{\pi }^{D\mathrm{/2}}}\int \frac{d\tau }{{\tau }^{D\mathrm{/2}-1}}\exp [-2\varepsilon \tau -{ {\mathcal E} }^{2}\frac{{\tau }^{3}}{12}].$$

Returning to the physical units, the total electric current density reads

22 $${J}_{x}=E\{{\sigma }_{n}+\frac{\omega T{\sigma }_{GL}}{{2}^{D}{\pi }^{D\mathrm{/2}}{T}_{c0}}\int \frac{d\tau }{{\tau }^{D\mathrm{/2}-1}}\exp [-2\varepsilon \tau -{(\frac{E}{{E}_{GL}})}^{2}\frac{{\tau }^{3}}{12}]\},$$

where *E*_*GL*_ was defined in Eq. (6) and *ω* is the dimensionless fluctuation stress parameter. The gap equation determining the dimensionless energy gap *ε* in this units is

23 $$\varepsilon =-\,\frac{1-T/{T}_{c0}}{2}+\frac{\omega T}{{2}^{D-2}{\pi }^{D\mathrm{/2}}{T}_{c0}}\int \frac{d\tau }{{\tau }^{D\mathrm{/2}}}\exp [-2\varepsilon \tau -{(\frac{E}{{E}_{GL}})}^{2}\frac{{\tau }^{3}}{12}].$$

### The dynamical instability point

**Figure 1:**
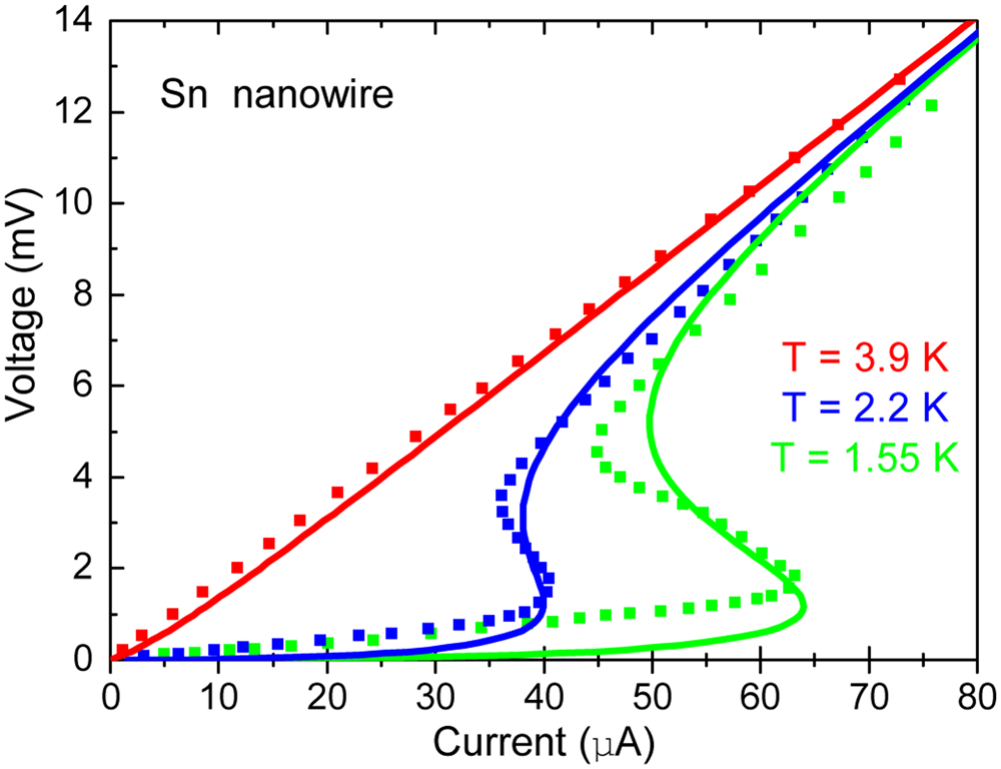
The *I*-*V* curves of 1*D* Sn nanowires^10^ at different temperatures. The points are the experimental data and the solid lines are the theoretical results.

The dynamical instability transition temperature on the phase diagram, T^*^, see Fig. 1, defined as a maximal temperature at which the instability appears. Mathematically is determined by vanishing of the first two derivatives, $\frac{d{J}_{x}}{dE}=0$ and $\frac{{d}^{2}{J}_{x}}{d{E}^{2}}=0$. Differentiating the current, Eq. (22) (via chain rule of the gap equation), results in:

24 $$\begin{array}{c}\,\frac{{\sigma }_{n}{T}_{c0}}{{\sigma }_{GL}{T}^{\ast }}+\frac{\omega }{{2}^{D}{\pi }^{D\mathrm{/2}}}\int \frac{d\tau }{{\tau }^{D\mathrm{/2}-1}}\exp [-2\varepsilon \tau -{(\frac{E}{{E}_{GL}})}^{2}\frac{{\tau }^{3}}{12}]\\ =\,{E}\frac{\omega }{{2}^{D}{\pi }^{D\mathrm{/2}}}\int \frac{d\tau }{{\tau }^{D\mathrm{/2}-2}}(2\frac{\partial \varepsilon }{\partial E}+\frac{{\tau }^{2}E}{6{E}_{GL}^{2}})\exp [-2\varepsilon \tau -{(\frac{E}{{E}_{GL}})}^{2}\frac{{\tau }^{3}}{12}];\end{array}$$

25 $$\int \frac{d\tau }{{\tau }^{D\mathrm{/2}-2}}\{\begin{array}{c}-\frac{E{\tau }^{2}}{2{E}_{GL}}+\frac{{E}^{3}{\tau }^{5}}{36{E}_{GL}^{3}}+{E}_{GL}\frac{d\varepsilon }{dE}(\frac{2{E}^{2}{\tau }^{3}}{3{E}_{GL}^{2}}-4)\\ +4\frac{E\tau }{{E}_{GL}}{({E}_{GL}\frac{d\varepsilon }{dE})}^{2}-2E{E}_{GL}\frac{{d}^{2}\varepsilon }{d{E}^{2}}\end{array}\}\exp [-2\varepsilon \tau -{(\frac{E}{{E}_{GL}})}^{2}\frac{{\tau }^{3}}{12}]=0$$

Now the dynamical instability transition temperature T^*^ is determined numerically taking into account the gap Eq. (18).

## Comparison with Experiments and Discussion

The results are first applied to a one dimensional superconductors - metallic wires and then for several qualitatively different types of 2D superconductors (as explained above, it is very difficult to observe the instability phenomenon in purely 3D materials, although in layered high *T*_*c*_ cuprates close to *T*_*c*_ the fluctuations become nearly 3D and the phenomenon was observed in magnetic field^17^.

### I-V curves of 1D Sn nanowires

We start with 1D nanowires. Granular superconducting *Pb* and *Sn* nanowires of quite regular cross - section and length have been produced by electro - deposition in nanoporous membranes^10^. It is important to note that the series of experiments of ref.^10^ on *Pb* and *Sn* nanowires is the only one (known to us) in which *both* the current and the voltage drives were employed. This allows a qualitative understanding of the important difference between the dynamical behaviour two. We focus on the voltage drive *I*-*V* curves of Sn.

The *I*-*V* curves, measured using the voltage drive at three temperatures, are shown by dotted lines in Fig. 1. The voltage drive employed clearly demonstrates the non - monotonic character below the onset *T*_c_ ≈ *T*_c0_ = 3.8*K* slightly above the bulk temperature of Sn (*T*_c_ = 3.72 K). An experiment on the same sample (see Fig. 3b in ref.^10^) demonstrates the voltage jumps over unstable domains of the dynamical phase diagrams. The jumps are more pronounced in Pb, see Fig. 3a of ref.^10^. This is consistent with the existence of the dynamical instability and was observed in numerous experiments (see 2D examples below).

The experimental data are fitted by Eqs (22, 23) for *D* = 1, see solid curves. The normal-state conductivity is given, *σ*_*n*_ = 3.6⋅10^4^ (Ω*m)^−1^, nanowires are 50 *μm* long with 55 *nm* in diameter. Measured material parameters are: coherence length^31^ *ξ* = 210 nm, penetration depth *λ* = 420 nm and the normal conductivity was obtained from the red doted line in Fig. 1. The values of fitting parameters are: the conductivity ratio *k* = *σ*_*n*_/*σ*_*GL*_ = 0.08 and the fluctuation strength parameter *ω* = 0.004, the value *ω* estimated from Eq. (5) is $\omega \simeq {10}^{-2}$.

This experiment has been already attempted to discuss in the framework of TDGL equations neglecting thermal fluctuations in ref.^9^, but only a qualitative explanation of S-shaped *V*(*I*) curves was achieved. Our approach yields the theoretical curves providing a fair agreement with the experiment and demonstrate that thermal superconducting fluctuations play the prime role in forming the instability.

### I-V curves of thin quasi-2D TiN films below *Tc*

The overheating model^1–4^ predicts that bistability occurs in *V*(*I*) at $T\lesssim {T}_{c}$. In experiment on thin films where the superconducting transition occurs in two stages^32^, the bistability develops at $T\lesssim {T}_{BKT}$^5^. Thus the naive thermal balance theory does not describe the switching behavior of a 2D system near *T*_*BKT*_ and in what follows we check if the experimental data can be reasonably fit by formulas of the inherent superconducting instability derived above. Before doing that, however, we present qualitative consideration indicating that in 2D the instability emerges at $T\le {T}_{BKT}$.

Now we turn to the analysis of experimental data. The measurements were taken on the titanium nitride (TiN) film having the thickness *d* = 7 nm, the sheet resistance in the normal state ${R}_{\square }=320$ Ω (*σ*_*n*_ = 4.3⋅10^4^ (Ω cm)^−1^) and the superconducting critical temperature *T*_*c*_ = 3.06 K. The film was formed on the Si/SiO _2_ substrate by the atomic layer deposition. Temperature dependence of the resistance of this film has been examined in^33^ where it was shown that the film is quasi-2D in the considered temperature range. The sample was patterned into bridges 50 *μ*m wide and 250 *μ*m long. Transport measurements are carried out using low-frequency ac and dc techniques in a four-probe configuration. The current drive was applied in a wide temperature range.

**Figure 2:**
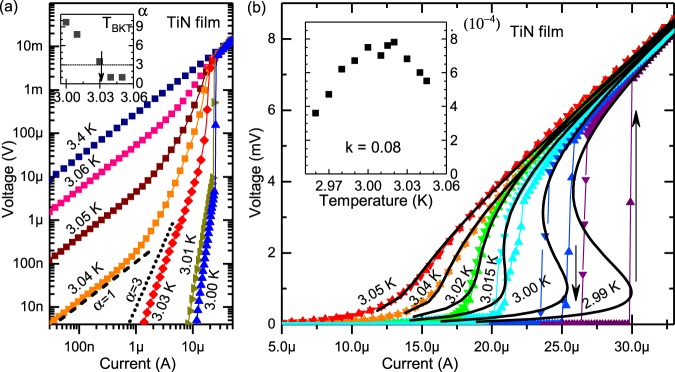
The *I*-*V* curves of TiN thin film at different temperatures. (**a**) The *I*-*V* curves shown in double logarithmic scale. Dotted line corresponds to *α* = 3 in *V* ∝ *I*^*α*^ dependence, dashed line corresponds to *α* = 1. Inset: temperature dependence of *α* exponent. Arrow marks the BKT transition temperature defined from the condition *α* = 3, ${T}_{BKT}\simeq 3.03$ K. (**b**) The *I*-*V* curves are shown in the linear scale. Solid lines are the theoretical curves according to Eqs (22, 23). Arrows mark the direction of the voltage jump: with the current increasing from zero–jump up; with the current decreasing to zero–jump down. Inset: The temperature dependence of the fitting parameter *ω*. At $T\simeq {T}_{BKT}$ parameter *ω* reaches the maximum.

We identify the BKT transition to superconducting state from the analysis of *V*(*I*) curves (see Fig. 2a) by the jump in the exponent *α* in the $V\sim {I}^{\alpha }$-dependence, *α* = 1 ↔ *α* = 3. Upon further cooling down, the bystabilily of *V*(*I*) curves with respect to applied current (see arrows if Fig. 2b) develops and we observe voltage jumps between the low-resistive and high-resistive states. The *I*-*V* dependencies calculated using Eqs (22, 23) for *D* = 2, for different temperature are shown in Fig. 2b as solid curves. The experimental data are fitted best with *T*_c0_ = 3.065 *K* and *k* = 0.08 which is close to *k* = *σ*_*n*_/*σ*_*GL*_ = 0.05, where we take *σ*_*GL*_ = 2*πc*^2^*ξ*^2^/*Dλ*^2^ (see Eq. 10) and *D* is the diffusion constant *D* = 0.7cm^2^/sec^5,33^; *ξ* = 8.2 nm; *λ*_*d*_ = 0.6 mkm. The fitting parameter is *ω* = 4⋅10^−4^ ÷ 8⋅10^−4^ (see inset in Fig. 2b) which is in agreement with *ω* ≈ 5⋅10^−4^ estimated from Eq. (5). One further sees that in the low voltage region fitting is expectedly worse since in the nonlinear response regime the superconducting fluctuations are practically absent. The values of the parameters *ω* and *k* were determined from fitting of experimental *V*(*I*) at currents *I* > *I*_*c*_, i.e. in the regions of instability and linear response. The linear resistance at currents *I* > *I*_*c*_ is practically independent on temperature. Since this linear response is determined by *σ*_*n*_, we assume that *k* does not depend on temperature as well. The parameter *ω* that characterizes the fluctuation strength (5) demonstrates the maximum around $T\simeq {T}_{BKT}$, which is natural since fluctuations are expected to enhance around the transition temperature^24^. The observed instability gets very quickly suppressed by the magnetic field (see Supplementary). A simple qualitative picture one can think of is as follows. If the magnetic field suppresses superconductivity, it should suppress the superconducting fluctuations as well.

### Layered materials

Instability in the form of the voltage jumps was observed recently in FeSeTe thin film on Pb(MgNb)TiO substrate^11^. Only the current drive was used, so that the S-shaped I-V curved cannot be determined. Only the voltage jumps were observed close to *T*_*c*_. The thickness of FeSeTe thin films is 200 nm. The layer distance *L*_*z*_ = 0.55 nm^34^. Here, the normal-state conductivity is taken to be *σ*_*n*_ = 1.3⋅10^4^(Ω*cm)^−1^.

**Figure 3:**
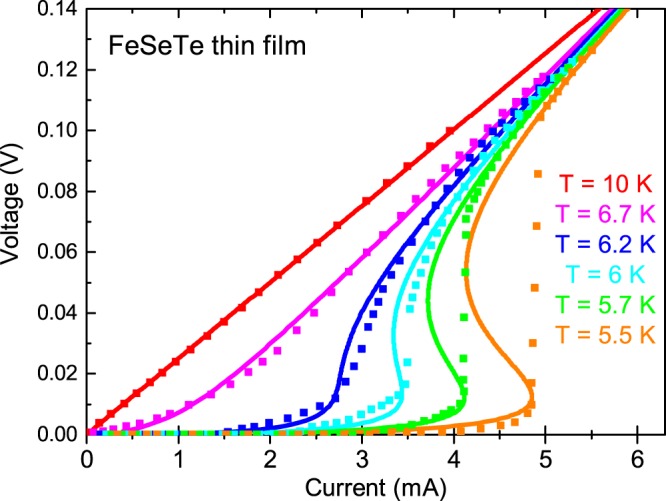
The I-V curves of *FeSeTe* thin film^11^ at different temperatures. The points are the experimental data and the solid lines are the theoretical fitting results.

The calculated *I*-*V* curves of the 2D FeSeTe thin film with different temperature are shown in Fig. 3 as solid curves. The experimental data of FeSeTe in a current driving setup from ref.^11^ are fitted best by the following values of parameters: *T*_c0_ = 7*K*, *k* = *σ*_*n*_/*σ*_*GL*_ = 0.07 and the fluctuation strength parameter *ω* = 0.01.

As well as in TiN film (Fig. 2b), the theoretical *I*-*V* curves for FeSeTe predict a re-entrant behavior for $T\lesssim {T}_{BKT}\approx 6$ K (at $T\lesssim 6$ K the low-voltage part of the *V*(*I*) curve is *V* ∝ *I*^*α*^, where *α* ≈ 3 at 6 K and increases with cooling down^11^). This re-enrance of *V*(*I*) is hard to observe directly in the current driving experimental setup. Experiments show that the current driven measurements lead to the “jump” in the *I*-*V* curves and the voltage driven measurements lead to the re-entrant S-Shaped *I*-*V* curve in the superconducting nanowires at low temperature^9^.

### Other layered materials

The instability in the ultra-thin granular YBa_2_Cu_3_O_7−*δ*_ nanobridges was clearly observed in a series of works in ref.^35^. Unfortunately a 2D or a 3D model cannot quantitatively describe these *I*-*V* curves since the fluctuations in this layered material and the temperature range can be described by a more complicated Lawrence - Doniach model. The generalization is possible but was not attempted in the present work.

Also, the “jump” *I*-*V* curves in a current driven setup was also reported in BSCCO^14^ that is clearly 2D. Unfortunately, *I*-*V* curve at the zero magnetic field was measured only at one temperature (76 K for *T*_*c*_ = 85.2 K).

## Conclusion

In this paper, *I*-*V* curve of a D-dimensional superconductor including the thermal fluctuations effects is calculated in arbitrary dimension using the dynamical self consistent gaussian approximation method. It is shown how the thermal fluctuations generate the S-shaped instability of *I*-*V* curves. The results are applied to analyse the transport data on various materials that possess sufficiently strong fluctuations in 1D or 2D. While it is found that the unstable region can exist also in 3D, the S-Shaped *I*-*V* curve in realistic materials show only in 1D superconductors.

## Electronic supplementary material

Supplementary Information:
