# Supplementary material for: Dynamical instability of the electric transport in superconductors
Source: Sci Rep. 2018 Sep 20;8:14104. doi: 10.1038/s41598-018-32302-8 (PMC6147792; doi:10.1038/s41598-018-32302-8)
Supplement: Supplementary file 1 — Supplementary Information: [file 41598_2018_32302_MOESM1_ESM.pdf]

Supplementary Information:

Dynamical instability of the electric transport in superconductors

Lei Qiao,[1,2] Dingping Li,[1,2] Svetlana V. Postolova,[3,4] Alexey Yu. Mironov,[4,5] Valerii Vinokur,[6] and Baruch Rosenstein[7,8]

[1]*School of Physics, Peking University, Beijing 100871, China*
[2]*Collaborative Innovation Center of Quantum Matter, Beijing, China*
[3]*Institute for Physics of Microstructures RAS, Nizhny Novgorod GSP-105, 603950, Russia*
[4]*A. V. Rzhanov Institute of Semiconductor Physics SB RAS, Novosibirsk, 630090, Russia*
[5]*Department of Physics, Novosibirsk State University, Novosibirsk, 630090, Russia*
[6]*Argonne National Laboratory, Materials Science Division, Lemont IL, 60439, USA*
[7]*Electrophysics Department, National Chiao Tung University, Hsinchu 30050, Taiwan, R. O. C*
[8]*Physics Department, Bar-Ilan University, 52900 Ramat-Gan, Israel*

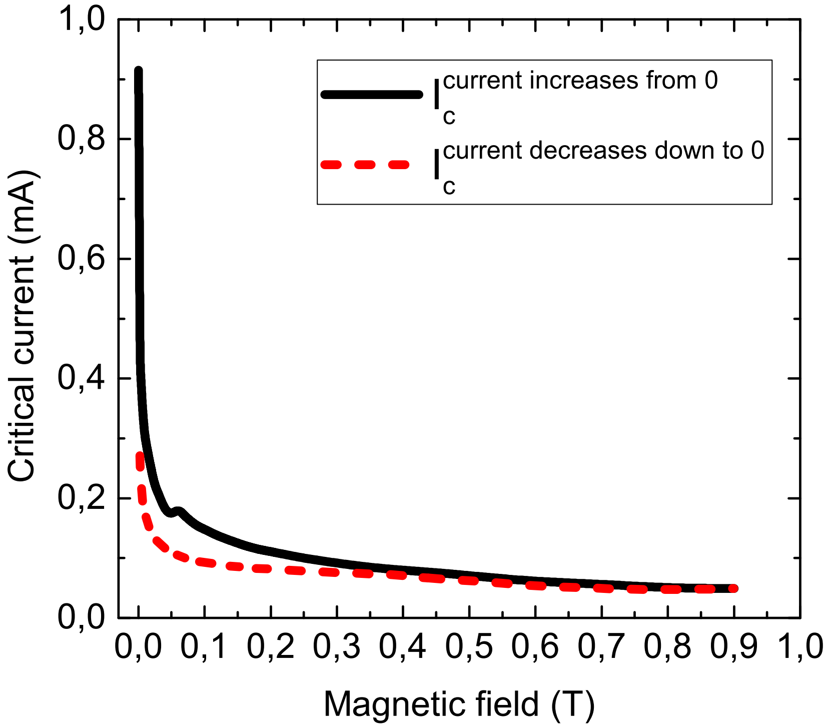

Figure S 1: Suppression of V(I) hysteresis by magnetic field. Critical current vs. applied perpendicular magnetic field. Data taken at T=200 mK. Here, critical current is the current at which system has voltage jump up to resistive state as current increases from $I = 0$ (solid line); current at which system has voltage jump down to superconducting state as current decreases down to $I = 0$ (dashed line). The instability means that these two critical currents do not match. As we see, the instability is suppressed very rapidly (the critical field for this sample is $B_c \approx 3$ T). We think that it demonstrates that the prime reason of the instability are superconducting fluctuations.
